# Excel for data visualization in academic health sciences libraries: a qualitative case study

**DOI:** 10.5195/jmla.2020.749

**Published:** 2020-01-01

**Authors:** Fred Willie Zametkin LaPolla

**Affiliations:** Research and Data Librarian, NYU Health Sciences, and Liaison, Departments of General Internal Medicine and Radiology, New York University Langone Health (NYU Langone), New York, NY, fred.lapolla@med.nyu.edu

## Abstract

**Background:**

Data visualization is a growing topic of discussion and area of educational programming in health sciences libraries. This paper synthesizes information on eight institutions’ experiences in offering Excel-focused data visualization workshops with the goal of providing an overview of the current state of educational offerings in this area.

**Methods:**

Semi-structured interviews were conducted by phone and email with librarians at institutions that offer Excel-focused workshops, which were identified by reviewing the websites of Association of Academic Health Sciences Libraries members and the 2019 Medical Library Association annual meeting program.

**Results:**

Librarians from six institutions were interviewed, online class materials from one institution were reviewed, and information from the author’s institution was included, resulting in a total of eight institutions. Educational offerings in Excel-focused data visualization ranged from one workshop to five workshops in a series, which typically first presented information for beginners and then progressed to more advanced data visualization skills. Regarding motivations for offering these workshops, librarians stated that they were committed to providing instruction in software programs that were already familiar to users. Workshop evaluations, when available, were generally positive.

**Discussion:**

Because of its widespread availability and usage, Excel offers a compelling opportunity for providing hands-on data visualization instruction in health sciences libraries.

## BACKGROUND

Data visualization is an increasingly common area of service in academic health sciences libraries [1, 2]. While the health sciences library literature includes discussions of instruction in specialized bioinformatics tools [3] and statistical coding tools like R [4, 5], less has been written about instruction in software with lower barriers to entry [6]. This paper aims to fill a gap by providing a high-level view of the current state of education about Excel-focused data visualization at multiple institutions.

Excel is a widely used, broadly available software tool that is often provided for free by employers for researchers and office workers. Given its ubiquity and familiarity among library users, it provides an opportunity for data visualization and data services instruction with relatively low upfront infrastructure costs and a perceived low barrier to entry.

“Perceived” is emphasized here because often users and instructors may feel that because they are familiar with Excel for storing data and simple analysis, it would also be easy to use for visualization. In reality, creating visualizations can often be more complex in Excel than in other tools, and less familiarity and comfort with Excel’s advanced features can heighten challenges for users. Fortunately, a cottage industry of thought leaders who provide tips and tricks for creating compelling data visualizations in Excel has sprung up [7–9], allowing librarians who are interested in providing data visualization services to learn an array of best practices and skills from a robust body of training materials.

This paper describes a qualitative case study examining Excel-focused data visualization instruction that is provided in academic health sciences libraries. The purpose of this research was to examine library programming from a broader scope than the individual library level with an eye on broader trends in the health sciences library sphere. By examining multiple institutions, the goal was to highlight trends in the burgeoning field of data visualization services in academic health sciences libraries.

## METHODS

Academic health sciences libraries that offer Excel-focused data visualization workshops and promote them on their website were identified. Criteria for inclusion in the environmental scan were being listed as a member library of the Association of Academic Health Sciences Libraries (AAHSL) and being located in the United States or Canada [10]. The library website of each US or Canadian AAHSL institution was visited, and site materials were reviewed to identify information on Excel workshops. Also, a Google search of “[the school’s or medical center’s site url]: excel” (e.g., “https://hms.harvard.edu: excel”) was performed to find any resources missed in the manual review of library websites. Libraries that offered either Excel workshops in general or Excel workshops specifically marketed in terms of data visualization were included. The website review was carried out between December 2018 and January 2019. Institutions offering Excel-focused data visualization workshops, as identified in 2019 Medical Library Association (MLA) annual meeting programming, were also included.

Librarians in institutions that were identified as providing Excel-focused workshops were contacted by email and telephone. Semi-structured interviews were employed to discuss their programming with the goal of uncovering common features. Prepared interview questions pertained to the intended audience of workshops, the time allotted for Excel instruction, the type of instruction (e.g., hands-on workshops versus other types of instruction), the topics or skills covered, and the general goals or purpose of the workshops. Interviewees were later followed up with via email to ask if they had workshop evaluation data, but these data were not obtained in all cases.

## RESULTS

Of 155 AAHSL institutions, 9 (6%) and 39 (25%) institutions had information online about Excel-focused data visualization workshops or general Excel-focused workshops, respectively. Two additional institutions were identified as providing Excel-focused data visualization workshops through presentations at the 2019 MLA annual meeting [6], bringing the total to 11 institutions. Librarians at 9 of these 11 institutions were contacted, excluding the author’s institution as well as 1 institution that offered the workshop through its Information Technology Department.

Librarians from six institutions responded. As a librarian from the University of Houston was not successfully contacted, relevant information was extracted by reviewing online class materials. Information from the author’s institution was also included. The eight institutions included in this study were New York University Langone Health (NYU Langone), Penn State University, Temple University, University of California, San Francisco (UCSF), University of Central Florida (UCF), University of Houston, University of Pittsburgh, and Wake Forest University. A description of each institution’s offerings is shown in Table 1.

**Table 1:**
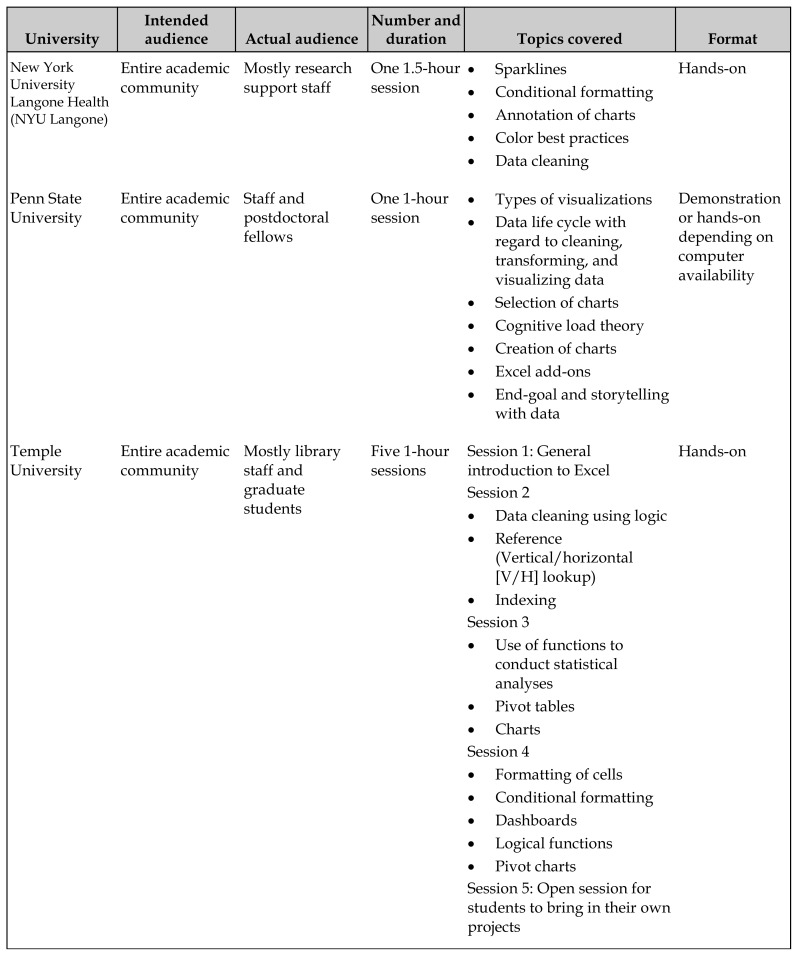

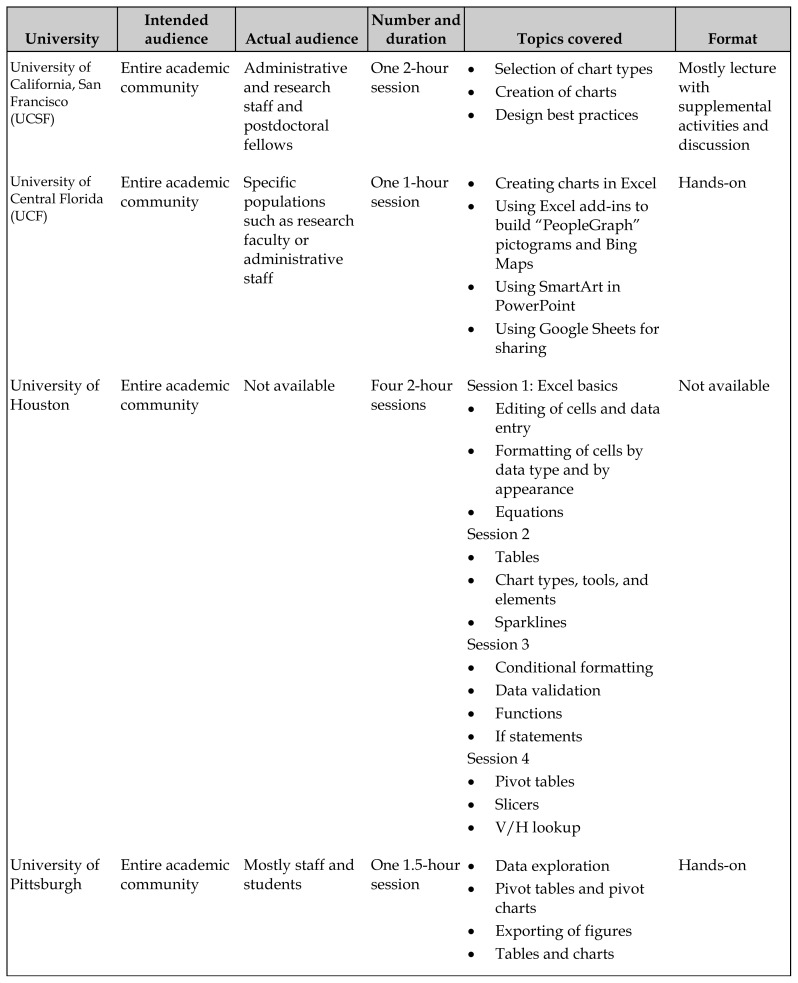
Description of Excel-focused data visualization workshops offered by eight institutions

University	Intended audience	Actual audience	Number and duration	Topics covered	Format
New York University Langone Health (NYU Langone)	Entire academic community	Mostly research support staff	One 1.5-hour session	Sparklines Conditional formatting Annotation of charts Color best practices Data cleaning	Hands-on
Penn State University	Entire academic community	Staff and postdoctoral fellows	One 1-hour session	Types of visualizations Data life cycle with regard to cleaning, transforming, and visualizing data Selection of charts Cognitive load theory Creation of charts Excel add-ons End-goal and storytelling with data	Demonstration or hands-on depending on computer availability
Temple University	Entire academic community	Mostly library staff and graduate students	Five 1-hour sessions	Session 1: General introduction to Excel Session 2 Data cleaning using logic Reference (Vertical/horizontal [V/H] lookup) Indexing Session 3 Use of functions to conduct statistical analyses Pivot tables Charts Session 4 Formatting of cells Conditional formatting Dashboards Logical functions Pivot charts Session 5: Open session for students to bring in their own projects	Hands-on
University of California, San Francisco (UCSF)	Entire academic community	Administrative and research staff and postdoctoral fellows	One 2-hour session	Selection of chart types Creation of charts Design best practices	Mostly lecture with supplemental activities and discussion
University of Central Florida (UCF)	Entire academic community	Specific populations such as research faculty or administrative staff	One 1-hour session	Creating charts in Excel Using Excel add-ins to build “PeopleGraph” pictograms and Bing Maps Using SmartArt in PowerPoint Using Google Sheets for sharing	Hands-on
University of Houston	Entire academic community	Not available	Four 2-hour sessions	Session 1: Excel basics Editing of cells and data entry Formatting of cells by data type and by appearance Equations Session 2 Tables Chart types, tools, and elements Sparklines Session 3 Conditional formatting Data validation Functions If statements Session 4 Pivot tables Slicers V/H lookup	Not available
University of Pittsburgh	Entire academic community	Mostly staff and students	One 1.5-hour session	Data exploration Pivot tables and pivot charts Exporting of figures Tables and charts	Hands-on
Wake Forest University	Entire academic community	Mostly students and staff	Four 1.5- to 2-hour sessions	Session 1: Basic Excel skills Modification of worksheets Formatting of worksheets Calculations Organization and visualization of data Printing Security Session 2: Advanced Excel skills Management of multiple worksheets Use of functions to conduct statistical analyses Management of worksheet functionality (validation and macros) Session 3: Data visualization Pivot tables and charts Slicers Sparklines Column, pie, and line charts Session 4: Formulas and functions Financial Logical Text Date and time Lookup Math and trigonometry	Hands-on

Most institutions for which data could be obtained indicated that their workshops were intended for their entire community (i.e., faculty, students, and staff) rather than specifically targeted to one group. A librarian at UCSF noted that their workshop was intended primarily for research and administrative staff and postdoctoral researchers. Multiple respondents said more staff and students tended to come to workshops, but a librarian from UCF said that they held workshops explicitly for faculty as part of a faculty development event. Librarians at both UCF and Penn State University indicated that their classes attracted a high number of administrative support staff who had been tasked by research faculty to help create figures for papers or slides.

Whereas three libraries offered multiple workshops in a series, five provided single stand-alone workshops. All workshops ranged from one to two hours in duration. Temple University provided the most extensive training, consisting of five sessions: a general introduction; focused sessions on data cleaning, data analysis, and complex visualizations including dashboards; and a final session for participants to bring in their own work and receive feedback. Wake Forest University offered four sessions: two two-hour sessions and two one-and-a-half-hour sessions. University of Houston also offered four sessions, which guided users from relatively introductory information on editing worksheets and using formulas to culminating in advanced Excel skills such as pivot tables and lookup functions that can be used in dynamic charts.

Of the five institutions that provided a single workshop, the University of Pittsburgh stood out for providing a deep exploration of Excel’s advanced analysis features, particularly pivot tables and pivot charts, which can be used to quickly summarize data to help uncover trends [11]. NYU Langone and Penn State University offered workshops focused primarily on chart creation. UCF provided a beginner-oriented workshop on creating charts and using add-ins for data visualization in Excel as well as using PowerPoint for “SmartArt” text visualizations and Google Sheets for sharing data. UCSF’s workshop focused on general best practices in data visualization with a discussion of Excel as one option for visualizing data.

Librarians at each institution had different motivations for teaching Excel. A librarian from Temple University noted that their goal was to impart skills to users (i.e., Excel vocabulary) to get them to a place where they could independently continue their learning journeys. A librarian at Temple University stated that they initially expected their workshop to be aimed at advanced Excel users but found significant demand for beginner-level instruction. Similarly, a librarian from Penn State University noted that one of their workshop goals was to build user confidence, but that the gap in user confidence with using Excel for visualization only became apparent once the workshop was offered and participants expressed a lack of confidence.

A librarian at UCF explained that their workshop was intended to help people present data in a way that makes salient points stick, to help faculty provide data visualization instruction to their students, and to help university affiliates represent the institution in a professional manner to stakeholders and outside organizations. Librarians from Wake Forest University, University of Pittsburgh, Penn State University, and NYU Langone provided variants on the theme that they offered instruction in Excel because it was widely used at their institutions and there was demand for this type of instruction.

Most libraries provided hands-on training to allow users to perform the data visualizations being discussed, though UCSF’s class was more lecture-based with activities throughout the class. Librarians at Temple University and UCF created space for students to demonstrate their learning by bringing in their own projects or uploading their work to a Google Sheet for sharing with the class. A librarian at Penn State University said that their workshop varied between a demonstration or a hands-on workshop, depending on the availability of computers in the classroom and whether participants brought their own laptops.

Workshop evaluation data could not be obtained from all institutions and were difficult to compare between institutions due to differences in methodological approaches. A librarian from Temple University indicated that formal evaluation was not conducted, but that instructors used a question-and-answer period at the conclusion of workshops to gauge their success (i.e., if students’ questions reflected understanding of the content). A librarian from UCF indicated that workshop feedback was positive. This institution also stood out for building skills assessment into the workshop in the form of having attendees upload and share a Google Sheet featuring their work.

Librarians at NYU Langone conducted surveys at the conclusion of each workshop and found that 100% of attendees who completed evaluation forms reported definitely or probably using what they learned, 93.8% found the level of material “just right,” and 96.9% would either “highly recommend” or “recommend” the workshop to others. UCSF’s workshop was also highly rated, with 91% of attendees stating the course was “excellent” or “very good.” Open-ended responses indicated that attendees appreciated the workshop format, which was based around “storytelling,” although some requested more hands-on work [8].

Librarians at the University of Pittsburgh indicated that their class was highly enjoyed, with all attendees rating it “excellent,” “very good,” or “good.” Many workshop attendees at this institution indicated that learning about advanced features, such as pivot tables, was the most helpful portion of the workshop. Other institutions did not provide evaluation data.

## DISCUSSION

As data visualization services become more common in health sciences libraries, educational workshops on available tools offer one of many possible routes for institutions to become involved in data visualization. This study shows that several libraries currently offer workshops focused on using Excel for data visualization, but often these workshops also teach skills related to data management and analysis. These workshops span from stand-alone sessions to five-session immersive experiences in the Excel environment. Most interviewees provide instruction at a level designed for relatively inexperienced Excel users but then build up to teaching more advanced data visualization, cleaning, and analysis skills. Thus, librarians who are interested in developing their own Excel-focused data visualization classes should consider starting with basic Excel skills and should not assume that users are familiar with advanced Excel functions. Furthermore, the workshops cover a mix of topics and levels of material, indicating that the topic of Excel is sufficiently flexible to allow librarians with beginner or advanced skills to provide meaningful data visualization education for their communities.

In addition to best practices in data visualization, several workshops included instruction on data cleaning and analysis, such as splitting columns, using “lookup” and other Excel functions, and creating pivot tables. This reflects the reality that users seldom, if ever, perform data visualization in isolation and that different phases of collection, cleaning, and analysis are interrelated with the process of visualizing data, which can be done to both analyze and communicate findings [2, 12]. Thus, some degree of competence in data cleaning and analysis would help data visualization librarians be able to speak meaningfully to the data-related skills needed to create figures for presentation, and a recent study found that one third of the libraries in their sample currently offer services in cleaning and wrangling data [12].

The topic of research reproducibility has attracted growing interest in scientific and popular publications in recent years, and this concern has extended to health sciences libraries [13, 14]. A drawback to using Excel is that it does not provide easily reproduced code or syntax that would make creation of charts reproducible, in contrast to tools such as SPSS and R Studio. To conduct reproducible research with Excel, the onus is on researchers to heavily document their processes, order of operations, and decision making, which may require a degree of description that is undesirable or impractical for most researchers. Thus, it could be beneficial for librarians to introduce other tools that may be more ideal for conducting reproducible research or to point researchers toward tools such as macros for Excel that perform repeatable actions [15].

The issue of reproducibility highlights a tension between teaching best practices and teaching skills that a research community requests. Providing instruction in tools that are already familiar to users may be better than exclusively focusing on the “best” tools, especially as it is unrealistic to expect all users are willing to learn more complex tools. Framed in a slightly different light, educational theorists Malcolm Knowles et al.’s theory of andragogy states that adults are motivated to learn when they have a need for information or skills in their lives [16]. Thus, data visualization librarians should help Excel users acquire data visualization skills to clearly and effectively communicate their work, while also encouraging their future engagement in more advanced data-related education.

Moreover, as revealed in the case of Penn State University’s class, individuals can also gain confidence in their skills by taking a data visualization workshop, which may in turn encourage further learning progressively outside of the learner’s original comfort zone. These classes could include bioinformatics or statistical tool–oriented classes that currently occur in the library community [3–5], and the provision of Excel-focused data visualization education need not be at the expense of more specialized tools or tools that provide more reproducible results.

It should also be noted that while Excel has limitations in regard to research reproducibility, given its widespread availability, it offers advantages from a data-sharing standpoint in that files can easily be shared with others and are unlikely to become obsolete in the near-term. Furthermore, Excel skills would not be institution-dependent, which may be the case with licenses to tools such as SPSS or SAS.

### Limitations

One limitation of this study is that some academic medical libraries might not post all of their educational offerings on their websites, particularly in a persistent way that is unconnected with temporary calendar announcements. As such, institutions that offered Excel-focused workshops were likely missed. Furthermore, the eight institutions described here may be atypical; thus, the present findings may have limited generalizability to the broader library community.

Using the AAHSL members list provided a pragmatic, bounded sample pool, but it is important to note that the distinction between academic medical libraries and general academic libraries may be more theoretical than actual. However, the benefits of exploring the services offered at academic institutions with a health sciences library may outweigh any drawbacks of making a false distinction between library types, especially as data visualization is a relatively new field for health sciences librarianship. While some institutions were undoubtedly missed by the author’s review of online materials, the approach of contacting AAHSL members can be considered to be more rigorous than relying on a strictly convenience sample.

Another limitation of this study is its uneven collection of evaluation data. The primary aim of the study was to provide a snapshot of current educational offerings in Excel-focused data visualization at health sciences libraries. However, given the vast differences between medical centers (e.g., dispersed campus medical systems versus urban academic medical centers versus hospital systems), librarians, ultimately, must judge what type of assessment is most valuable to their individual contexts, regardless of what is performed elsewhere.

A final limitation is that data visualization services are still at a burgeoning phase in academic health sciences libraries. Although the present findings may quickly become outdated, they are meant to provide a snapshot of current practices so that other librarians can gain insight into and build upon the work being done at other institutions.

Providing instruction in data visualization using Excel is being approached in different ways by academic health sciences libraries. A common theme is that many librarians seek to teach skills beyond data visualization, including those related to data cleaning and analysis. These findings suggest that librarians who support data visualization may also need to have some degree of competence in other data-related skills. However, given the ubiquity of Excel and its perceived low barrier to entry for users, Excel-focused data visualization instruction can provide an avenue for providing new services that library users desire.

## 

**Fred Willie Zametkin LaPolla**, fred.lapolla@med.nyu.edu, https://orcid.org/0000-0002-3185-9753, Research and Data Librarian, NYU Health Sciences, and Liaison, Departments of General Internal Medicine and Radiology, New York University Langone Health (NYU Langone), New York, NYs
